# The molecular identity of the TLQP-21 peptide receptor

**DOI:** 10.1007/s00018-021-03944-1

**Published:** 2021-10-09

**Authors:** Bhavani S. Sahu, Megin E. Nguyen, Pedro Rodriguez, Jean Pierre Pallais, Vinayak Ghosh, Maria Razzoli, Yuk Y. Sham, Stephen R. Salton, Alessandro Bartolomucci

**Affiliations:** 1 National Brain Research Centre, NH-8, Manesar, Gurugram, Haryana 122052 India; 2 Department of Integrative Biology and Physiology, University of Minnesota, 2231 6th St. SE, Minneapolis, MN 55455 USA; 3 Bioinformatics and Computational Biology Program, University of Minnesota, Minneapolis, USA; 4 Departments of Neuroscience and Geriatrics and Palliative Medicine, Friedman Brain Institute, Icahn School of Medicine at Mount Sinai, One Gustave L. Levy Place, New York, NY 10029 USA

**Keywords:** G-protein-coupled receptor, VGF, TLQP-21, C3aR1, C3a, gC1QR, HSPA8, Complement, Microglia, Adipocytes

## Abstract

The TLQP-21 neuropeptide has been implicated in functions as diverse as lipolysis, neurodegeneration and metabolism, thus suggesting an important role in several human diseases. Three binding targets have been proposed for TLQP-21: C3aR1, gC1qR and HSPA8. The aim of this review is to critically evaluate the molecular identity of the TLQP-21 receptor and the proposed multi-receptor mechanism of action. Several studies confirm a critical role for C3aR1 in TLQP-21 biological activity and a largely conserved mode of binding, receptor activation and signaling with C3a, its first-identified endogenous ligand. Conversely, data supporting a role of gC1qR and HSPA8 in TLQP-21 activity remain limited, with no signal transduction pathways being described. Overall, C3aR1 is the only receptor for which a necessary and sufficient role in TLQP-21 activity has been confirmed thus far. This conclusion calls into question the validity of a multi-receptor mechanism of action for TLQP-21 and should inform future studies.

## Introduction and scope of the review

The murine *Vgf* gene (non-acronymic) encodes for a 617-amino acid (615 in human)-long pro-protein and peptide precursor, classified as a member of the extended granin family [1]. VGF is cleaved by prohormone convertases such as PC1/3 and PC2 to generate low-molecular weight peptides which are secreted through the regulated pathway [1, 2]. The 21-amino acid-long TLQP-21 is arguably the most studied and best characterized among the VGF-encoded peptides [3, 4]. After its identification in the rodent brain [3], sympathetic nerves [5] and sensory neurons [6], it emerged as a pleiotropic neuropeptide involved in various physiological processes such as lipolysis, microglial activation, pain, sexual and depression-like behavior and energy balance [1, 7–10]. However, most of our knowledge on the pharmacological and physiological role of TLQP-21 derives from pharmacological gain of function experiments pre-dating the identification of its cognate receptor. To date, three binding targets, have been proposed for TLQP-21, namely Complement 3a Receptor 1 (C3aR1) [11], the receptor for the globular head of the complement protein C1q (gC1qR) [12], and the Heat Shock Protein Family A member 8 (HSPA8) [13].

Here, we aim to provide a critical review of the molecular identity of the TLQP-21 receptor as well as to evaluate the hypothesis that multiple receptors are responsible for TLQP-21 biological activity.

Upon critical evaluation of experimental evidence available to date, sequence alignment of pharmacophores and analysis of mode(s) of binding of receptor ligands, we conclude that C3aR1 is the only cognate receptor of TLQP-21 for which a necessary and sufficient role has been confirmed, and a molecular mechanism has been proposed for its activation upon ligand binding. Additionally, a multi-receptor mechanism of action is not supported by experimental results. This conclusion should be taken into account when designing future mechanistic studies as well as in drug discovery and development experiments focused on this promising therapeutic target for human diseases.

## The TLQP-21 neuropeptide

*Identification, biochemistry and evolution.* TLQP-21 was originally identified in the rodent brain using mass spectrometry [3]. Matrix-assisted laser desorption/ionization-time of flight (MALDI-TOF) mass spectrometry (MS) analysis of immunoprecipitates of VGF peptides from rat brain allowed the identification of a pseudomolecular ion at a mass to charge (*m/z*) of 2433.53 designated as TLQP-21 corresponding to positions 556–576 of the rat VGF sequence. The consequent analysis identified the sequence as TLQPPASSRRRHFHHALPPAR. Its name derives from the first four N-terminal amino acid sequence (Thr-Leu-Gln-Pro) combined with the specification of its length, following standard convention in VGF biology [2]. TLQP-21 derives from the cleavage of the precursor TLQP-62 [3, 14], although the exact biochemical steps for this proteolytic cleavage have not been clarified yet. Alanine-scanning mutagenesis, molecular modeling and NMR studies corroborated the identification of the pharmacophore of TLQP-21 as the three C-terminal residues PAR [4, 15].

A recent evolutionary protein sequence analysis of TLQP-21 for the mammalian peptide showed that 96% of mammals had a positively charged C-terminal residue [4] with the human sequence being the ancestral and evolutionarily conserved sequence, while a unique gain of function mutation emerged in two subfamilies of rodents (see below for details on the pharmacology). All primates express a C-terminal PSR motif which is also the most dominant motif representing 56% of the sequences analyzed [4]. On the other hand, *Muridae* and *Cricetidae* (subfamilies of rodents including mice, rats, and hamsters) express the PAR motif at the C-terminus. As will be discussed below, pharmacological evidences suggest that this S to A mutation may represent a gain of function enabling small-sized rodents to have a selective advantage to mobilize energy [4].

### Tissue distribution and regulation of expression

Due to intrinsic biological constraints (a peptide encoded by a pro-peptide precursor) and limitations in current bioassay development/validation, there is limited knowledge on the tissue distribution and regulation of TLQP-21. The expression of the *Vgf* gene has been used as a proxy of TLQP-21 expression. Studies of VGF mRNA described prominent expression in the central and peripheral nervous system and in several neuroendocrine glands including the pancreas, adrenal medulla and pituitary [1]. However, since VGF encodes for at least 8 biologically active peptides generated through cell and organ-dependent proteolytic cleavage in the secretory granules [1, 2], VGF gene expression should not be used as a unambiguous measure of TLQP-21 regulation/expression and investigators should use proteomics to confirm the presence of the peptide cleavage products. Similarly, the presence of “TLQP- motif containing peptides” has been described in enterochromaffin-like cells, somatostatin cells, pancreas and the hypothalamus [8, 16–18]. It must be noted that the antibodies used against the N-terminus of TLQP-21 do not discriminate among TLQP-21 and other peptides containing the “TLQP” sequence. Recently, a selective rabbit anti-mouse TLQP-21 has been generated [5], allowing the identification of expression in sympathetic nerves innervating the adipose tissue [5] and sensory neurons [6]. However, its use has been limited thus far, and no comprehensive organ mapping has been conducted. Finally, a few experimental [16, 17] or commercially available enzyme-linked immunosorbent assay (ELISA) kits for TLQP-21 are available. While, they provide valuable insights, unfortunately they are limited in scope due to partial validation or significant cross-reactivity with overlapping VGF fragments including the TLQP-62 precursor, a peptide for which a cognate receptor has not been identified yet and which shows a pharmacology often inconsistent with TLQP-21. Indeed, while TLQP-21 and TLQP-62 treatment resulted in overlapping, but not identical, reduction in neuropathology in 5xFAD model of Alzheimer`s disease [19] and thermal and mechanical hypersensitivity [20], several studies showed non-overlapping and potentially opposing effects of either the two peptides or non-cross reactive antibodies directed specifically to TLQP-21 or TLQP-62, including in: contextual fear conditioning memory [21], behavior in depression-related tests like the forced swim test and social interaction test [22] and the firing rate of hypothalamic neuronal networks [23]. Studies in insulinoma [14] and CHO cells [24], revealed different potencies of the two peptides, and moreover, stimulation of insulin secretion by TLQP-62 in insulinoma cells was not blocked by the C3aR1 antagonist SB290157 [14]. Overall, the preponderance of non-overlapping biological functions of TLQP-21 and TLQP-62 suggests that assays which detect significant overlap between these two (and potentially other) VGF-derived peptides containing the TLQP motif can have limited validity to discriminate TLQP-21 expression. Technological innovation and systematic studies are desperately needed to address these gaps of knowledge.

### Biological role of TLQP-21: in vitro studies

The best characterized second messenger elicited by TLQP-21 treatment in various cell lines is calcium mobilization [4, 11, 12, 25–28]. The source of calcium, i.e., extracellular vs intracellular, as well as the signaling cascade(s) downstream of calcium influx seems to vary among cell lines—although little direct cross cellular comparison has been attempted thus far.

One of the best characterized cellular functions of TLQP-21 is the potentiation of adrenergic-induced lipolysis in adipocytes [4, 5, 15, 28]. Specifically, TLQP-21 is not a lipolytic molecule per se but dose-dependently increases βAR (adrenergic receptors)-induced lipolysis with a mechanism leading to calcium influx from transient receptor potential cation channels (TRPC) from the extracellular compartment and downstream activation of CaMKII/ERK1/2 (calcium/calmodulin-dependent protein kinase II/Extracellular signal-regulated kinases) [4, 5, 28]. While this pathway is insufficient to mediate hormone sensitive lipase (HSL) activation and cause lipolysis, it potentiates phosphorylation of HSL elicited by βAR activation, causing a sustained pro-lipolytic effect. Interestingly, it was shown that the mouse TLQP-21 (mTLQP-21) sequence is more potent than the human TLQP-21 (hTLQP-21) sequence in potentiating adrenergic-induced lipolysis in both rodent and human adipocytes [4], a result consistent with the lower potency of hTLQP-21 reported for other assays [4, 11, 15] and its evolutionary history (see above). As discussed below, albeit the human and mouse peptides have different potency in most (but not all [29]) assays, the molecular pathway is conserved and the mouse peptide has been proposed an ideal template for translational studies and drug design [4, 15].

Another cellular model that has been often used to probe TLQP-21 activity is microglia [29–31]. TLQP-21 increases the phagocytic potential of microglia via the uptake of fibrillar amyloid-β (fAβ) or fluorescently labeled beads, as well as by increasing cell migration in a wound healing assay [29]. One study in particular showed that TLQP-21 impairs microglial P2Y-mediated purinergic signaling while modulating phagocytic activity [30].

Aside from adipocytes and microglia, other studies confirmed a functional role for TLQP-21 in other cell types including: neuroprotection in cerebellar granule cells [25]; a trophic effect on GH3 cell line [27]; and finally, enhancement of β-cell survival and glucose-stimulated insulin secretion [9].

### Biological role of TLQP-21: in vivo studies

TLQP-21 has been implicated in various physiological processes including but not limited to metabolic regulation [3, 9, 28, 32, 33], nociception [6, 34], sexual behavior [35], anxiety/stress-related behavior [36, 37] (In this review, we will only highlight the major physiological functions of TLQP-21. Readers are directed to systematic review papers for a comprehensive discussion on the biological role of this neuropeptide [1, 38–40]).

The first report on the biological role of TLQP-21 derived from chronic intracerebroventricular (icv) infusion in mice, whereby the peptide increased resting energy expenditure and rectal temperature, coupled with prevention of diet-induced obesity [3]. Molecular analyses suggested that this effect is mediated by activating the sympathetic outflow pathway to adipose fat pads [3]. Follow-up studies confirmed this initial observation in mice and hamsters [3, 8, 18, 32, 41]. In contrast to mice, TLQP-21 reduces food intake in Siberian hamsters. Chronic peripheral TLQP-21 injection decreases body weight and fat mass in diet-induced obese mice without changes in food intake or, surprisingly, energy expenditure measured with indirect calorimetry [28], as well as in hamsters where hypophagia is also observed [18]. The TLQP-21-induced anti-obesity effect is prevented by germline C3aR1 knockout in mice [28]. Moreover, experiments conducted on lean hamsters through TLQP-21 administration exposed to short photoperiods caused reduced food intake, while the same was not seen in hamsters exposed to long photoperiods [18, 32]. At variance, a recent report showed no anti-obesity effect of TLQP-21 in diet-induced obese mice [23]. Several methodological differences must be noted among these studies, including the duration of high-fat diet (HFD) (9 [28] vs 32 [23] weeks) and peptide infusion (28 [28] vs 11 days [23]). Furthermore, in the recent study [23], but in no previous study, TLQP-21 was dissolved in Polysorbate-80 (Tween-80) a solvent known to cause inflammation and metabolic syndrome [42] and to increase C3a activation (the originally identified ligand for C3aR1) [43, 44]; overall, this suggests a potential interference of Tween-80 with the mechanism of action of TLQP-21 which remains to be investigated.

Another prominent function of TLQP-21 is to regulate pain behavior and nociception. In mouse models of nerve injury and inflammation, VGF expression was shown to drastically increase in the spinal cord and dorsal root ganglia neurons [45]. Intraplantar injection of TLQP-21 leads to hyperalgesia in rodent models of inflammatory pain induced by formalin [34], while TLQP-21 elicits an analgesic response when administered icv in the same pain model [34]. On the other hand, intrathecal TLQP-21 injection elicits hyperalgesia and contributes to nerve injury-induced hypersensitivity with a mechanism that requires C3aR1 expression in microglia and is mediated by calcium influx and p38 activation [6, 7]. While the mechanism for this differential effect has not been firmly established yet, it is hypothesized that the central effect of TLQP-21 can be due to activation of downstream pathways regulating pain behavior, while peripheral injection could directly activate the receptor in nerve terminals and/or local immune cells activating an inflammatory response.

In parallel to a prominent function of TLQP-21 on microglia (discussed above), a recent study demonstrated that icv infusion of TLQP-21 can reduce the amount of amyloid plaques present in brain parenchyma, improving overall neuropathology in the 5xFAD mouse model of Alzheimer’s disease [29].

Overall, the versatile biology of TLQP-21 in functions as diverse as lipolysis, neurodegeneration and metabolism, suggests an important role in several human diseases.

## The identity of the TLQP-21 receptor

Pharmacological and biophysical studies conducted before the first receptor was identified in 2013 suggested a unique binding site for TLQP-21 in 3T3-L1 and CHO cells [5, 26]. Furthermore, two independent studies used ^125^I-TLQP-21 or ^18^F-JMV5656 (a TLQP-21 derivative) to explore the in vivo binding and distribution of these peptides upon intravenous (iv) injection [46, 47]. High binding in pancreas, liver, adipose, kidney, adrenal glands and heart was observed, with negligible binding in the brain, indicating that the peptide does not cross the blood–brain barrier when peripherally injected. Although these studies did not directly probe the binding in knockout models for the proposed TLQP-21 receptors, they indirectly support the existence of cells expressing the receptors in those organs.

**Fig. 1:**
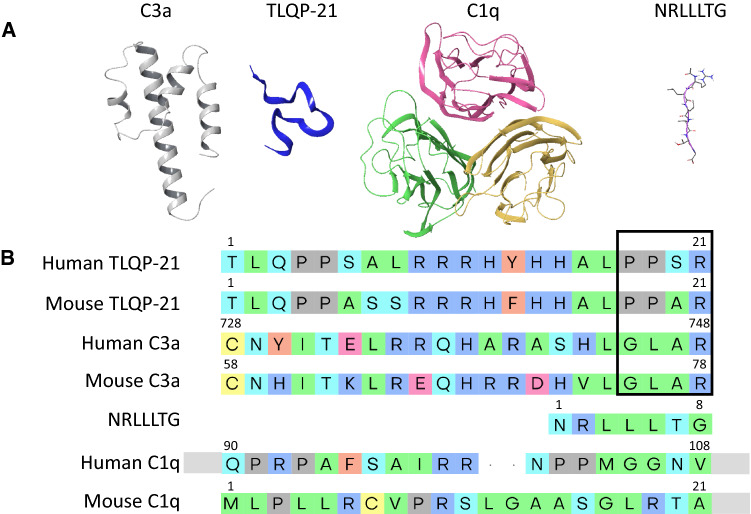
Best characterized ligands for C3aR1, gC1qR and HSPA8. **A** Left to right: three-dimensional structures of human C3aR1 (PDB (Protein Data Bank https://www.rcsb.org): 4HW5 [78]), TLQP-21 (XP_011514851.1, homology modeled from sequence), NRLLLTG peptide (PDB: 4PO2 [79]), human C1q (PDB: 6FCZ [74]). Structures are shown as ribbon representation and colored by chain and ribbon with sticks for NRLLLTG. **B** Multiple sequence alignment of human and mouse variants of: TLQP-21, C3a, C1q, and HSPA8 ligand NRLLLTG. Alignment performed with MUSCLE in Schrödinger Multiple Sequence Viewer (Maestro, Schrödinger, LLC, New York, NY, 2020) with 10.0 opening gap and 0.20 extending gap penalties and manually inspected. Only sequence regions aligning to TLQP-21 (21 residues) are shown for simplicity. Gray rectangles indicate extension of sequence not shown. Black outline indicates TLQP-21 and C3a residues indicated as pharmacophores

**Fig. 2:**
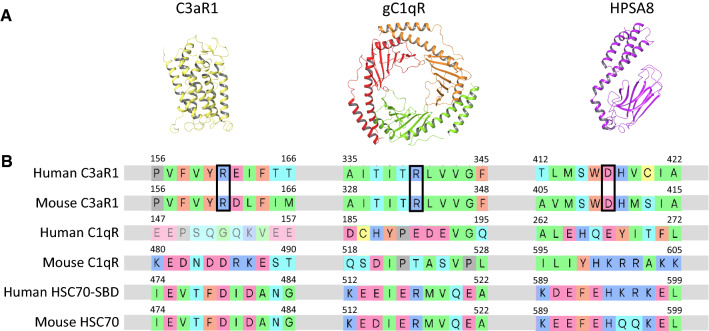
Structure and sequence of C3aR1, gC1qR and HSPA8. **A** Left to right: three-dimensional structures of human C3aR1 (NP_001313406.1, homology model [4]), C1qR (PDB: 6SZW [80]), and structure binding domain of HPSA8 (PDB: 4PO2 [79]). Structures are shown as ribbon representation and colored by chain. **B** Multiple sequence alignment of human and mouse sequences of C3aR1, C1qR, and HSPA8. Alignment performed with MUSCLE in Schrödinger Multiple Sequence Viewer (Maestro, Schrödinger, LLC, New York, NY, 2020) with 10.0 opening gap and 0.20 extending gap penalties and manually inspected. Only sequence regions aligning to TLQP-21 (21 residues) are shown for simplicity. Gray rectangles indicate extension of sequences not shown. Black outline indicates C3aR1 residues that interact with pharmacophore

Three putative binding targets of TLQP-21 have been identified. The first receptor identified was C3aR1 [11], with gC1qR [12] and HSPA8 [13] being described shortly thereafter (Figs. 1 and 2). The following sections critically review the published evidence in support of each of these targets and evaluate the notion of multiple receptors and mechanisms responsible for TLQP-21 biological activity.

### Identification of C3aR1 as the TLQP-21 receptor

C3aR1 is a 7-transmembrane G-protein-coupled receptor (GPCR) that was first identified as the receptor for the complement activation product C3a [48] (Fig. 2). C3aR1 is expressed in various tissues including adipose, brain and pancreas with higher expression seen in high-fat diet fed obese rodents [4, 28, 46, 49–51]. The predominant cell type in which C3aR1 is expressed are macrophages and other immune cells, while expression in other stromal cells and adipocytes has also been reported [4, 28, 46, 49–51]. Regarding expression in the CNS, multiple studies have shown robust C3aR1 expression in microglia [7, 29–31, 52].

The C3aR1 molecular pharmacology is incompletely understood. In general, ligand-mediated C3aR1 activation leads to Gi/o protein activation and βarrestin1/2 recruitment [4, 7, 51, 53, 54]. However, a few studies also suggest the activation of alternative G proteins (e.g., Gs, Gq) [4, 7, 51, 53, 54] and a number of downstream signaling pathways, among which increased intracellular calcium is the most commonly reported [4, 11, 24, 51, 52, 55–58].

There is currently no resolved 3D structure of C3aR1. Human C3aR1 (hC3aR1) shares approximately 57% sequence homology to C5aR1, whose structure was previously solved by X-ray crystallography and which provided the first glimpse of the orthosteric binding site and was used as a template for homology modeling of C3aR1 [4, 59].

Experiments conducted on CHO-K1 and O-342 cell lines have led to the identification of C3aR1 as the TLQP-21 receptor [11]. Following its original identification, an independent study confirmed the identity of C3aR1 as the TLQP-21 receptor using photoaffinity labeling of N-terminal biotin-conjugated TLQP-21 peptides in membranes from CHO and 3T3-L1 cells [15].

### Evaluation of C3aR1 as the TLQP-21 receptor

RNA-Seq screening of putative GPCRs, pharmacological experiments and siRNA screens were used by Hannedouche and co-workers to confirm the necessary role of C3aR1 for TLQP-21 mediated biological effects [11]. Furthermore, NMR studies demonstrated that TLQP-21 undergoes a disorder-to-order transition from random coil to α-helix in the presence of cells expressing C3aR1, an effect that is absent in C3aR1 KO cells and can be antagonized pharmacologically [15]. In the same study, the TLQP-21 pharmacophore was identified at its C-terminus using Alanine-scanning mutagenesis. Mutations in the last four amino acids caused a progressive decrease in functionality, while mutation or amidation of the C-terminal Arginine blocks the biological activity and receptor activation [15]. Furthermore, molecular modeling studies showed the C-terminal -AR motif to interact and form salt bridges with 3 residues in the mouse or human C3aR1: R161, R340 (R333 in mouse), and D417 [4]. This mode of binding is consistent with C3a binding to C3aR1, highlighting a conserved mechanism of ligand-mediated receptor activation [60]. Sequence analysis demonstrated that the dominant C-terminal motifs of C3a and TLQP-21 both contain a terminal—AR (Fig. 1). Amongst 82 mammalian sequences examined, 71% expressed the C-terminal—AR motif for C3a [4]. Overall, these data suggest that the C-terminal arginine of C3a is the pharmacophore for C3aR1 activation, forming a salt bridge at D417. Consistently, site-directed mutagenesis of D417 and other key binding site residues (R161 and R340) results in inability of C3a to bind and activate C3aR1 [4, 53, 61]. Interestingly, in addition to the two endogenous ligands also the SB290157 and JR14a contain a chemically modified uncapped Arginine [62], further supporting the requirement of this motif for C3aR1 binding and suggesting that the remaining portion of the peptides or small molecules confers specificity and regulates signaling and μM or nM potency.

Overall, pharmacological and structural analyses demonstrate that C3a and TLQP-21 are both full agonists at C3aR1 [4, 11] (This review focuses primarily on studies relevant for the identification of TLQP-21 receptor, the reader is referred to excellent reviews discussing details of C3aR1 and C3a pharmacology and physiology [51, 60, 63, 64]). A systematic and extensive pharmacological comparison between C3a and TLQP-21 has not been performed yet, and the signaling activated by TLQP-21 has only been investigated in a handful of studies and cell lines. However, the two peptides can pharmacologically antagonize each other [15], and elicit similar physiological effects in adipocyte and microglia cell lines [28, 29], with C3aR1 expression being require for biological function of the two ligands [4, 49, 52]. Specifically, several studies used in vitro and/or in vivo genetic loss of function approaches or used C3aR1 antagonists to probe the role of this receptor on TLQP-21 biological activity. TLQP-21 activates microglia to induce calcium mobilization [7], uptake of fibrillary amyloid-β [29] and, finally to regulate inflammatory pain [7]. Two independent studies demonstrate that knocking down (KD) C3aR1 using siRNA in the BV2 microglial cell line [31] or microglia from C3aR1-deficient mice [29], result in decreased basal phagocytosis, prevents TLQP-21 induced uptake of fibrillary amyloid-β and prevents TLQP-21 evoked calcium mobilization. It was also observed that the differentially expressed genes upon TLQP-21 administration were abrogated by C3aR1 deletion in primary microglia [29]. Furthermore, C3aR1 KD using shRNA in 3T3L1 prevents TLQP-21-induced calcium influx, substrate phosphorylation and pro-lipolytic activity [4]. It should be noted that, similar to several other cases of loss of function of GPCRs [65], C3aR1 KD adipocytes manifested increased basal and isoproterenol-induced lipolysis [4], suggesting that a comprehensive multifactorial experimental approach should be used to fully understand the role of C3aR1 in ligand-mediated lipolysis. Finally, and consistent with the effect seen in adipocytes, C3aR1 knockout prevented the TLQP-21-induced anti-obesity effect seen in wild-type mice [28].

Although mode of binding, mechanism of receptor activation and signaling mediated by C3a or TLQP-21 are largely conserved, the two peptides are also known to have different physiology. Indeed, TLQP-21 exerts a predominantly "beneficial" metabolic and neurological effect [9, 28, 29, 66] while C3a exerts a predominantly "negative" proinflammatory and anaphylactic-like effect [51, 52, 58, 61, 63]. The source and mechanism of this differential effect remain to be established.

The C3aR1 antagonist SB290157 [67] has been shown to antagonize several TLQP-21 biological effects in cultured primary microglia, microglia cell lines, mast cells or 3T3-L1 cells [4, 7, 30, 31, 62]. For example, TLQP-21-evoked potassium currents in microglia cells were blocked by SB290157 inhibition with only 1/11 cells tested showing a small TLQP-21 response in the presence of the antagonist [30]. However, SB290157 also exerts an agonist effect on C3aR1-mediated functions such as calcium influx, β-lactamase activity and ERK signaling [57, 68]. Evidence that SB290517 can act both as an antagonist and an agonist depending on cellular context suggests that experiments based only on SB290157 to infer (or exclude) a role for C3aR1 on TLQP-21 induced effects should be considered with caution. Other C3aR1 antagonists have been proposed [61, 62], but the most potent molecules, e.g., JR14a family of compounds, are derivative of SB290157, and their validation is thus far limited.

To the best of our knowledge, only two studies report data which are partially inconsistent with a necessary role of C3aR1 for TLQP-21 induced effect. In the first study, partial C3aR1 KD obtained by using siRNA did not reduce calcium influx elicited by the TLQP-21 derivative JMV5656 (TLQP-21 was not used in this study) [69]. However, the alternative mechanisms to C3aR1 activation were not investigated. In a second study, the evidence was presented that an anti-C1qBP monoclonal antibody mAB-C1qBP (see below for the detailed review on the role of gC1qR) but not SB290157, inhibits TLQP-21-induced inhibition of ATP-evoked outward potassium currents in microglia [30]. This evidence has been used to suggest that C3aR1 mediates some, but not all functions of TLQP-21. The concerns on the exclusive use of SB290157 to rule out a role for C3aR1 have been discussed above. Furthermore, the use of an antibody against a receptor to infer ligand-mediated activation and its impact on downstream signaling, in the absence of independent verification of its specificity and function blocking activity, provide less rigorous support for the activation of gC1qR by TLQP-21, compared to C3aR1.

**Fig. 3:**
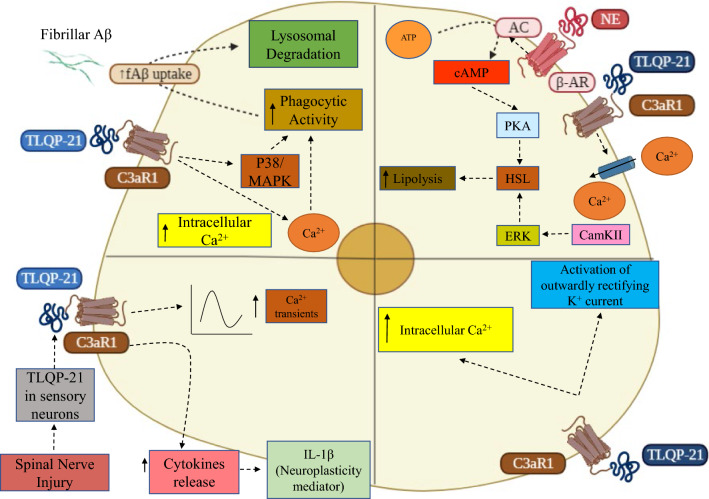
TLQP-21 mediates intracellular signaling via C3aR1 activation in different cell types. Top left: BV2 microglia cells (modified from [29, 31]).TLQP-21 mediates P38 Mitogen Activated Protein Kinase (P38/MAPK) pathway through Complement C3a Receptor 1(C3aR1) causing an increase in phagocytic activity leading to increased fibrillar amyloid β (fAβ) uptake leading to lysosomal degradation. Top right: adipocytes (modified from [4, 5, 28]). TLQP-21 mediates β Adrenergic Receptor (β-AR)-induced lipolysis in a C3aR1-mediated fashion by intracellular calcium mobilization and MAPK/Extracellular Regulated Kinase (ERK) activation of Hormone Sensitive Lipase (HSL). Bottom left: Spinal cord microglia (modified from [7]). TLQP-21 mediates through C3aR1 an increase in calcium transients in spinal cord microglia as well as an increase in cytokine release and IL-1β release in a pronociceptive manner as well as for spinal neuroplasticity following nerve injury. Bottom right: primary microglia (modified from [30]. TLQP-21 mediates in a C3aR1-dependent pathway, an activation of membrane currents, Ca2 + responses and stimulation of migration and phagocytic activity in microglia. *AC* Adenylate Cyclase, *ATP* Adenosine Tri-Phosphate, *cAMP* Cyclic Adenosine Monophosphate, *PKA* Protein Kinase A, *NE* Norepinephrine

Overall, a critical assessment of published loss of function or pharmacological experiments from multiple laboratories, provide substantial evidence for a necessary and sufficient role of C3aR1 to explain the biological activity TLQP-21 (summarized in Fig. 3).

### Identification of gC1qR as the TLQP-21 receptor

gC1qR, also known as p33, is a cellular homotrimer protein of 33 kDa that was identified as a receptor for the globular head of the complement protein C1q [70] (Fig. 2). gC1qR is produced as a pre-protein and assembles into a donut-shaped homotrimer with acidic ligand and cell-binding surfaces [70]. gC1qR is found predominantly in the mitochondrial matrix and in cellular compartments such as the endoplasmic reticulum, the nucleus, and at the cell surface in some cells [71]. Classic studies demonstrated that the globular head of gC1qR is secreted in response to immune cell activation and it can be considered to be a regulator of complement activation [72]. The original ligand identified for gC1qR, C1q is a 460 kDa hexameric protein that is the first recognition subcomponent in the classical complement pathway [73, 74] (Fig. 1). C1q is formed from 18 polypeptide chains (2 each of chains A, B, and C) that form a ‘bouquet of flowers-like structure’ consisting of a collagen-like stem domain and 6 globular flower heads which are important for ligand recognition [74].

Chen and co-workers identified gC1qR as an alternative receptor for TLQP-21 using affinity chromatography and mass spectrometry-based approaches [12]. The study utilizes a chemically modified TLQP-21 consisting of a biotinylated label added to the N-terminus of the peptide with an extended cysteine residue at the C-terminus, cross-linked to sulfo-EMCS (N-*ε*-maleimidocaproyl-oxysulfosuccinimide ester). Incubation of the modified biotin-TLQP-21-C-sulfo-EMCS peptide enables covalent binding to membrane proteins from the brain and spinal cord of adult rats, and protein separation using avidin monomeric column. Bottom-up proteomics analysis by nano-LC–MS/MS analysis of the eluents identified three distinct fragments belonging to the gC1qR [12]. It should be noted that TLQP-21 binding to and activation of C3aR1 requires an uncapped C-terminus [15]. The study by Chen and co-workers did not report the presence of C3aR1. Thus, it is possible that the addition of a cysteine residue to the C-terminus of TLQP-21 with a chemically reactive cross-linker favored binding to gC1qR and impacted the binding to and identification of C3aR1.

### Evaluation of gC1qR as the TLQP-21 receptor

To the best of our knowledge, only two studies directly tested the role of gC1qR in TLQP-21-mediated effects. After identifying gC1qR as a putative receptor for TLQP-21, Chen and co-workers used siRNA or incubated macrophages with a monoclonal antibody against gC1qR to demonstrate inhibition of TLQP-21-mediated effects [12]. The increase in intracellular Ca2 + levels elicited in macrophages by TLQP-21 was attenuated by either siRNA or neutralizing antibodies against gC1qR. Similarly, a neutralizing antibody directed against gC1qR was used in rats following partial sciatic nerve ligation, resulting in a delayed onset of TLQP-21-mediated and nerve injury-associated mechanical hypersensitivity [12]. A separate study suggested that both C3aR1 and gC1qR could contribute to the function of TLQP-21 in modulating microglia [30]. However, in this latter study, only one experiment was provided to support a role for gC1qR in TLQP-21 signaling, while several other experiments proved a direct role for C3aR1 (see above). This experiment focused on TLQP-21-induced inhibition of ATP-evoked outward potassium currents. While SB290157 did not prevent the TLQP21-induced effect, preincubation of microglia with mAB-C1qBP blocked its effect [30]. Concerns about the mixed agonist/antagonist profile of SB290157 (see above) and the use of a blocking antibody against gC1qR suggest these potentially interesting findings should be verified by additional experimentation utilizing different loss of function approaches.

Another aspect should be considered in this critical assessment of available evidence supporting the identity of gC1qR as a TLQP-21 receptor. Sequence alignment of the chain A of C1q (from PDB: 6FZC) with m/hTLQP-21 show limited overall amino acid similarity, and no sequence similarity at the C-terminus of TLQP-21 (Fig. 1), which represents the peptide hot spot for its biological activity [4, 7, 15, 29].

Overall, considering published evidence to date, only one study has provided direct evidence in support of a necessary role of gC1qR for TLQP-21 activity. Other studies have provided some suggestive evidence of the involvement of this receptor in TLQP-21 activity, but these studies were mostly based on either unclear or negative outcomes using the C3aR1 antagonist SB290157 or the use of neutralizing antibodies against the gC1qR. This led us to conclude that strong support for gC1qR as a specific TLQP-21 receptor is lacking. The possibility exists that, similar to many other small peptides [70], TLQP-21 engages in non-selective binding to gC1qR.

#### Identification and evaluation of HSPA8 as the TLQP-21 receptor

HSPA8, a 646-amino acid-long protein, is known by many names, such as heat shock cognate 71 kDa protein, heat shock 70 kDa protein 8, HSC70, and HSP73 [75] (Fig. 2). HSPA8 is a constitutively and ubiquitously expressed protein, found both intracellularly, in the cytoplasm and nucleus and extracellularly on the cell membrane. One of its major cellular functions is in protein folding of newly synthesized polypeptides and reducing protein aggregation [75, 76]*.* Evidence points towards the presence of multiple binding sites of HSPA8 to various cofactors and ligands (e.g., NRLLLTG), conferring upon it a variety of functions. For example, EWI-2, an early activation marker of dendritic cells, was identified as a ligand of HSPA8 by binding to HSPA8-expressing cells and the immobilized HSPA8 protein [77].

Using affinity chromatography, mass spectrometry as well as molecular dynamics, a recent study suggested that hTLQP-21 binds to HSPA8 expressed on the plasma membrane of SH-SY5Y neuroblastoma cells [13]. Similar to the study identifying gC1qR as an alternative receptor for TLQP-21, this study also employed cysteine addition to the C-terminus of TLQP-21 (the peptide hot spot for biological function). Furthermore, docking and simulation study of the TLQP-21 bound homology model of HSPA8 was not followed up with experimental validation studies.

After its initial identification, no additional study has been published on the role of HSPA8 in TLQP-21 biological activity, and as a result, the significance of TLQP-21 binding to HSP8 cannot be fully evaluated at this time.

## Summary

Interest in the TLQP-21 neuropeptide has significantly increased after its original identification in 2006 [3], and following the recognition of multiple potential receptors or binding partners in subsequent years [11–13]. The existence of multiple putative binding partners for TLQP-21 requires the undertaking of a critical evaluation of the available evidence in support of this multi-receptor model. This is an important step in the mechanistic analysis of its mode of action and a full evaluation of its potential for pharmacotherapies.

Biophysical and biochemical data are inconsistent with multiple TLQP-21 binding sites [5, 24, 26]. Furthermore, structural/molecular determinants of TLQP-21 binding, the requirement for uncapped C-terminal arginine for its biological activity, and the evidence that C3aR1 deletion abrogates TLQP-21 induced activity in vitro and in vivo, conclusively support the identity of C3aR1 as a TLQP-21 receptor (e.g., [4, 7, 28]). Conversely, evidence in support of gC1qR and HSPA8 as specific cognate receptors is very limited thus far, based on the use of C-terminally modified peptides that can interfere with its biological function, and some of the experiments are based solely on the use of incompletely characterized neutralizing antibodies against the receptor to probe the signaling pathway. It is, thus, possible that binding of TLQP-21 to gC1qR could be non-specific since this receptor binds to a plethora of proteins and peptides found in plasma, on the cell surface and on microorganisms [70]. Similarly, HSPA8 is a molecular chaperone for several proteins and no loss or gain of function experiment has been published in relation to TLQP-21 activity.

Overall, based on experimental evidence published to date, we conclude that: 1) the only receptor for which sufficient experimental evidence has been obtained by multiple independent groups supporting its identity as the TLQP-21 receptor is the C3aR1; and 2) the multi-receptor model is not supported by independent replication of experimental data.

## Gaps of knowledge, limitations and future directions

The identification of C3aR1 as the TLQP-21 receptor is a major step forward for future studies aimed at mechanistically disentangling its biology as well as in probing its potential for translational and drug discovery and development.

Yet, gaps of knowledge and weaknesses in the existing literature were noted. First, the use of the putative C3aR1 receptor antagonist SB290157 remains problematic because of its reported partial agonist activity; further research should address its molecular mechanism(s) of action and attempts should be made to develop novel C3aR1 antagonists [62]. Second, there is an incomplete knowledge of TLQP-21 and C3aR1 expression (and a lack of specific reagents to assess it) [49], and it has been difficult to determine under which physiological conditions and in which organs this peptide and its receptor are regulated. Third, since TLQP-21 and C3a are two endogenous ligands of C3aR1, it is essential to define whether the signaling pathways they activate are similar, as well as to identify the conditions favouring local tissue availability of each of them. Fourth, how ligand-mediated C3aR1 receptor activation differs to cause similar but not fully overlapping physiological effects in vivo is not presently understood. Fifth, with the emergence of microglial C3aR1 as potentially the predominant TLQP-21-activated receptor in the CNS, future studies will need to determine how/whether this microglial signaling pathway contributes to the control of energy homeostasis, nociception, sexual behavior, anxiety/stress-related behavior, and the development and progression of neurodegenerative disease, bioactivities all demonstrated for TLQP-21. Finally, evidence suggesting that the TLQP-21/C3aR1 sequence co-evolved in mammals [4], with the human peptide/receptor system representing the ancestral form characterized by low pharmacological potency, should be addressed by follow-up experiments designed to fully investigate the potential of C3aR1 as a new target for human diseases.

## Data Availability

Not applicable.
